# Choroidal blood perfusion as a potential “rapid predictive index” for myopia development and progression

**DOI:** 10.1186/s40662-020-00224-0

**Published:** 2021-01-04

**Authors:** Xiangtian Zhou, Cong Ye, Xiaoyan Wang, Weihe Zhou, Peter Reinach, Jia Qu

**Affiliations:** 1 Eye Hospital and School of Ophthalmology and Optometry, Wenzhou Medical University, Wenzhou, Zhejiang China; 2 National Clinical Research Center for Ocular Diseases, Wenzhou, Zhejiang China; 3 State Key Laboratory of Optometry, Ophthalmology and Vision Science, Wenzhou, Zhejiang China; 4 Research Unit of Myopia Basic Research and Clinical Prevention and Control, Chinese Academy of Medical Sciences (2019RU025), Wenzhou, Zhejiang China

**Keywords:** Myopia, Choroidal thickness, Choroidal blood perfusion, Rapid predictive index

## Abstract

Myopia is the leading cause of visual impairment worldwide. The lack of a “rapid predictive index” for myopia development and progression hinders the clinic management and prevention of myopia. This article reviews the studies describing changes that occur in the choroid during myopia development and proposes that it is possible to detect myopia development at an earlier stage than is currently possible in a clinical setting using choroidal blood perfusion as a “rapid predictive index” of myopia.

## Background

Presently, repeated measurements of refraction and axial length over time are required to obtain a definitive clinical diagnosis of myopia progression. These “downstream indicators” do not facilitate early intervention to prevent myopia progressing into a more severe stage, which may even cause irreversible blindness. Establishing a “rapid predictive index” is of interest to ophthalmologists in order to improve both the management and prevention of myopia. Changes in choroidal structure and function are recently suggested to be contributory factors of myopia onset and progression, although the underlying mechanism is still not clear. Therefore, it is worthwhile to determine if changes in non-invasive choroidal measurements can be used to establish an index of this process.

## Main text

Myopia is the leading cause of visual impairment worldwide [1]. Its alarmingly rapid increase in prevalence has raised significant concerns regarding its impact on public health. The number of affected people is expected to triple from 1.41 billion with a prevalence of 22.9% in 2000 to 4.76 billion to reach 49.8% in 2050 [2]. Younger generations are getting myopic at an earlier age, especially in East Asian countries, including Singapore, Japan, Korea, and China [3, 4]. Meanwhile, the global prevalence of high myopia is also predicted to rise from 2.7% (163 million) in 2000 to a markedly elevated level of 9.8% (938 million) in 2050 [2]. Severe myopia increases the risk of irreversible blindness due to retinal detachment, macular degeneration, glaucoma, and choroidal neovascularization [1, 5, 6]. Furthermore, visual impairment resulting from both uncorrected myopia and myopia-related ocular complications heighten socioeconomic burdens. Annually, the direct cost of visual impairment caused by myopia is 755 million dollars in Singapore [7] and at least 3.8 billion dollars in the United States [8]. These alarmingly high costs accompanied estimated declines in the gross domestic product of 202 billion dollars worldwide [9]. The “myopia pandemic” has caught the attention of the World Health Organization, who advocates inclusion of myopia assessment into the general eye care service [10].

Myopia is a complex disease with a multi-factorial etiology whose underlying pathophysiological mechanisms are not yet fully understood. Two of its signature changes include excessive optical axis elongation, concurrent scleral thinning accompanying remodeling of its extracellular matrix [11–14]. These alterations result in declines in the visual acuity of distant images. This malfunction allegedly stems from aberrant transduced retinal signals excessively lengthening the optical axis along with the aforementioned changes in the scleral structure and content. Despite this realization, it is still unclear how visually-induced retinal signal transduction events elicit control of scleral and optical axis elongation during ocular development.

There is emerging evidence that myopia has risk factors involving interactions between environment and genetic risk factors. One of the environmental variables thought to influence myopia progression during development especially in school age children includes time spent performing near work [15, 16] and exposure to outdoor daylight [17, 18]. Some insight has been obtained from studies focused on clarifying how visually evoked signals at the retina are transduced and then induce molecular signaling pathway activation. These messages may regulate choroidal function and/or traverse into the sclera to affect changes that modulate responses described in experimental myopia animal models [19–21]. Such communication is required in order for the scleral stiffness to decrease and stretch to accommodate excessive optical axis elongation. Changes in choroidal thickness and function are also suggested to be contributory factors since it is juxtaposed between the retina and sclera. Secondly, the choroid may also act as a barrier to the diffusion of endogenous growth factors that promote axial elongation [22, 23]. Finally, the choroid itself may be one of the sources of factors that directly influence scleral stiffness and extensibility through control of its molecular content and turnover [24, 25]. Therefore, studies on functional changes that occur in the choroid during myopia development are relevant to clarifying the pathophysiological events driving this process in experimental animal models.

Clinicians commonly use time dependent measurements of refraction and axial length to evaluate myopia progression. Establishing with statistical analysis a definitive diagnosis, may require obtaining repeated measurements for periods over more than a year. Furthermore, during extended periods using these traditional metrics as outcome measurements in clinical myopia research may sometimes confound interpretation of the results due to contributions by extraneous factors. Such prolongation could also increase the likelihood that the study subjects may be exposed to procedures that are deemed ineffective in myopia control. Another concern is that sole reliance on measurements of refraction and axial length as the “downstream indicators” may not be able to resolve rapid progression of myopia in the clinic. Therefore, establishing a “rapid predictive index” will be invaluable in the management and prevention of myopia. Such an index would be an asset because it would provide an accurate evaluation of myopia susceptibility and the effects of therapeutic measures on its development. One possibility includes changes in axon myelination, which was reported as a biomarker for myopia development in the form deprivation myopia chicken model [26, 27]. There are also indications that evaluation of parameters characteristic of choroidal function may identify changes that can rapidly predict the likelihood of myopia onset and track the progression of this disease process.

### Choroidal thickness and myopia

With the advent of optical coherent tomography (OCT), cross-sectional viewing of the posterior segment of the eye became possible [28]. The results show that the retinal thickness is thinner in myopic subjects than in non-myopic subjects [29–32]. Recently, Fourier-domain OCT is increasingly being used to monitor changes in the choroid that occur during myopia development. With this innovation, the anterior and posterior boundaries of the choroid are identified in a cross-sectional image. Accordingly, choroidal thickness is defined as the vertical distance between the two boundaries at a specific location or over a defined area.

The Beijing Eye Study reported that the sub-foveal choroid shrank by 32 μm for every 1 mm increase in axial length in 3233 subjects [33]. These findings were supported by the subgroup analysis done in the Shandong Children Eye Study [34]. In the subgroup of 972 school children aged more than 6 years, they found that thicker sub-foveal choroid was associated with shorter axial length and it decreased by 25 μm per millimeter increase in axial length. Additionally, the Beijing Eye Study reported later that thicker peripapillary choroidal thickness was also associated with a shorter axial length in 3060 subjects [35]. In another cross-sectional analysis of the data from the Correction of Myopia Evaluation Trial (COMET) cohort, differences in choroidal thickness were found among different ethnicities after adjustment for axial length; namely, Asians had the thinnest choroidal thicknesses [36]. Given that myopia is more prevalent in Asian populations, thinner choroidal thickness might be a risk factor for myopia development.

Longitudinal designs were used to measure changes in choroidal thickness in myopia. Such an analysis is complex because of the confounding influence of age-related changes on choroidal thickness. Several studies found that in adults choroidal thickness became thinner with increasing age at rates ranging from 14 to 54 μm per decade [33, 37–39]. In contrast, choroidal thickness in children with no significant refractive errors increased with age [40–42], while in children with a wider range of refractive errors, age dependent changes in choroidal thickness were similar to the pattern observed in adults [43–45]. Read et al. [46] followed 41 myopes and 60 non-myopes aged between 10 and 15 years for 18 months and found that in children whose axial elongation increased more rapidly, choroidal thickening during this period was less than in other subjects undergoing more moderate and slower increases in axial elongation. Fontaine et al. [47] also found in a 15 months follow-up that sub-foveal choroidal thickness increased in non-myopic eyes, but it decreased in myopic eyes of 115 children aged from 2 to 16 years. In a 2020 study, Xiong et al. [48] followed a cohort of 756 participants with different refractive statuses aged from 6 to 18 years for 1 year and reported that age as well as refractive status could simultaneously influence the pattern of changes in choroidal thickness, which may explain why previous studies failed to detect such an association. Furthermore, this recent report also demonstrated that newly developed myopic subjects experienced more evident choroidal thinning compared with both non-myopic individuals and those afflicted with myopia for a longer period.

All of the above evidence supports the notion that choroidal thinning may be reflective of myopia development. However, even though several different animal myopia model studies obtained results in agreement with those reported in clinical studies [19, 21, 49], the mechanism was unknown that controls choroidal thickness. Further study is still needed to elucidate whether the changes occur in parallel or precede development of myopia. Furthermore, in the latter case, it is also uncertain how changes in the choroid thickness manifest such a major regulatory role on myopia development.

### Choroidal blood perfusion and myopia

Advances in OCT technology resulted in development of optical coherence tomography angiography (OCT-A), which makes it possible to non-invasively quantify choroidal vessel density [50, 51]. This innovation facilitates evaluating changes in choroidal vessel density in clinical and animal studies of myopia. Currently, there are only a few available OCT-A clinical studies since it is a relatively new imaging modality. Another limitation is that their conclusions are not consistent with one another. Milani et al. [52] found no significant difference in choriocapillaris perfusion area between 42 myopic and 40 control eyes in a retrospective study. In contrast, measuring instead a larger range of choroidal area and using another algorithm, Mo and colleagues [53] reported a marginally significant difference in choriocapillaris blood flow density between 45 emmetropic and 41 high myopic eyes. This finding agrees another study reported by Al-Sheikh et al. [30], in which the area of flow deficit in the choriocapillaris increased in eyes with greater myopia. In one of our recently published studies [54], the choroidal vascularity and choriocapillaris flow voids were measured and compared between eyes of 31 anisomyopic young adults. We found that choroidal vascularity and choriocapillaris blood flow were lower in more myopic individuals.

Despite the variability in measurement of choroidal parameters, either intra-individual or inter-device was found to be acceptable [55–57]. It is worth noting that confounding factors, such as age, systolic blood pressure and measurement time should be taken into consideration when analyzing choroidal thickness or blood perfusion change [58]. Meanwhile, with such small changes in choroidal parameters, it will be difficult to predict the onset and progression of myopia. However, this hurdle could be due to a current technical limitation that is likely to be lessened with the advent of a new device and updated algorithms.

Clinical evidence is still very inadequate to make any definitive conclusion regarding an association between changes in choroidal blood flow and myopia. Our recent studies may shed some light on this question that could ultimately help clarify the relationship between choroidal vascularity and myopia development. Wu et al. [12] found that scleral hypoxia is a key modulator of scleral remodeling during myopia development. Later, Zhang and associates [59] demonstrated that in guinea pigs with temporal ciliary artery transection, choroidal blood perfusion was significantly reduced from the baseline in the temporal quadrants, but not in the nasal quadrants. The choroidal thickness in the temporal but not the nasal quadrants also decreased, which was likely caused by the reduced choroidal blood perfusion. The same study found that choroidal blood perfusion and its thickness were both significantly reduced in spontaneous and three different experimental animal myopia models. Another recent study from our group showed that prazosin, a vasodilator, increased choroidal blood perfusion and inhibited both myopia progression as well as eyeball elongation via reducing scleral hypoxia in guinea pigs [60]. In view of all of the above findings, it is plausible that changes in choroidal thickness in myopia might result from reduced choroidal blood perfusion, which in turn may lead to scleral hypoxia. This condition could in turn trigger downstream receptor-linked signaling pathways events that induce responses promoting myopia progression.

## Conclusions

Taken together, the above considerations prompt us to hypothesize that measurements of choroidal blood perfusion have the potential to provide a sensitive and “rapid predictive index” of myopia onset. Further study is warranted to test the possibility that such measurements will make it possible to detect myopia development at an earlier stage than is currently possible in a clinical setting.

## References

[1] Saw SM, Gazzard G, Shih-Yen EC, Chua WH (2005). Myopia and associated pathological complications. Ophthalmic Physiol Opt.

[2] Holden BA, Fricke TR, Wilson DA, Jong M, Naidoo KS, Sankaridurg P (2016). Global prevalence of myopia and high myopia and temporal trends from 2000 through 2050. Ophthalmology.

[3] Ding BY, Shih YF, Lin LLK, Hsiao CK, Wang IJ (2017). Myopia among schoolchildren in East Asia and Singapore. Surv Ophthalmol.

[4] Morgan IG, Ohno-Matsui K, Saw SM (2012). Myopia. Lancet.

[5] Marcus MW, de Vries MM, Junoy Montolio FG, Jansonius NM (2011). Myopia as a risk factor for open-angle glaucoma: a systematic review and meta-analysis. Ophthalmology.

[6] Lavanya R, Kawasaki R, Tay WT, Cheung GCM, Mitchell P, Saw SM (2010). Hyperopic refractive error and shorter axial length are associated with age-related macular degeneration: the Singapore Malay eye study. Invest Ophthalmol Vis Sci.

[7] Zheng YF, Pan CW, Chay J, Wong TY, Finkelstein E, Saw SM (2013). The economic cost of myopia in adults aged over 40 years in Singapore. Invest Ophthalmol Vis Sci.

[8] Vitale S, Cotch MF, Sperduto R, Ellwein L (2006). Costs of refractive correction of distance vision impairment in the United States, 1999-2002. Ophthalmology.

[9] Fricke TR, Holden BA, Wilson DA, Schlenther G, Naidoo KS, Resnikoff S (2012). Global cost of correcting vision impairment from uncorrected refractive error. Bull World Health Organ.

[10] World Health Organization. The impact of myopia and high myopia: report of the Joint World Health Organization–Brien Holden Vision Institute Global Scientific Meeting on Myopia, University of New South Wales, Sydney, Australia, 16–18 March 2015. Geneva: World Health Organization; 2017. https://www.who.int/blindness/causes/MyopiaReportforWeb.pdf. Accessed 11 Dec 2020.

[11] Shih YF, Fitzgerald ME, Norton TT, Gamlin DP, Hodos W, Reiner A (1993). Reduction in choroidal blood flow occurs in chicks wearing goggles that induce eye growth toward myopia. Curr Eye Res.

[12] Wu H, Chen W, Zhao F, Zhou Q, Reinach PS, Deng L, et al. Scleral hypoxia is a target for myopia control. Proc Natl Acad Sci U S A. 2018;115(30):E7091–E7100. 10.1073/pnas.1721443115 PMC6064999 29987045

[13] Moriyama M, Ohno-Matsui K, Futagami S, Yoshida T, Hayashi K, Shimada N (2007). Morphology and long-term changes of choroidal vascular structure in highly myopic eyes with and without posterior staphyloma. Ophthalmology.

[14] Fitzgerald ME, Wildsoet CF, Reiner A (2002). Temporal relationship of choroidal blood flow and thickness changes during recovery from form deprivation myopia in chicks. Exp Eye Res.

[15] Saw SM, Chua WH, Hong CY, Wu HM, Chan WY, Chia KS (2002). Nearwork in early-onset myopia. Invest Ophthalmol Vis Sci.

[16] Ip JM, Saw SM, Rose KA, Morgan IG, Kifley A, Wang JJ (2008). Role of near work in myopia: findings in a sample of Australian school children. Invest Ophthalmol Vis Sci.

[17] Wu PC, Chen CT, Lin KK, Sun CC, Kuo CN, Huang HM (2018). Myopia prevention and outdoor light intensity in a school-based cluster randomized trial. Ophthalmology.

[18] Xiong S, Sankaridurg P, Naduvilath T, Zang JJ, Zou HD, Zhu JF (2017). Time spent in outdoor activities in relation to myopia prevention and control: a meta-analysis and systematic review. Acta Ophthalmol.

[19] Wallman J, Wildsoet C, Xu A, Gottlieb MD, Nickla DL, Marran L (1995). Moving the retina: choroidal modulation of refractive state. Vis Res.

[20] Wildsoet C, Wallman J (1995). Choroidal and scleral mechanisms of compensation for spectacle lenses in chicks. Vis Res.

[21] Hung LF, Wallman J, Smith EL (2000). Vision-dependent changes in the choroidal thickness of macaque monkeys. Invest Ophthalmol Vis Sci.

[22] Summers JA (2013). The choroid as a sclera growth regulator. Exp Eye Res.

[23] Nickla DL, Wildsoet CF, Troilo D (2001). Endogenous rhythms in axial length and choroidal thickness in chicks: implications for ocular growth regulation. Invest Ophthalmol Vis Sci.

[24] Marzani D, Wallman J (1997). Growth of the two layers of the chick sclera is modulated reciprocally by visual conditions. Invest Ophthalmol Vis Sci.

[25] Shelton L, Troilo D, Lerner MR, Gusev Y, Brackett DJ, Rada JS (2008). Microarray analysis of choroid/RPE gene expression in marmoset eyes undergoing changes in ocular growth and refraction. Mol Vis.

[26] Swiatczak B, Feldkaemper M, Schaeffel F (2019). Changes in fundus reflectivity during myopia development in chickens. Biomed Opt Express.

[27] Swiatczak B, Feldkaemper M, Schraermeyer U, Schaeffel F (2019). Demyelination and shrinkage of axons in the retinal nerve fiber layer in chickens developing deprivation myopia. Exp Eye Res.

[28] Huang D, Swanson EA, Lin CP, Schuman JS, Stinson WG, Chang W (1991). Optical coherence tomography. Science.

[29] Li M, Yang Y, Jiang H, Gregori G, Roisman L, Zheng F (2017). Retinal microvascular network and microcirculation assessments in high myopia. Am J Ophthalmol.

[30] Al-Sheikh M, Phasukkijwatana N, Dolz-Marco R, Rahimi M, Iafe NA, Freund KB (2017). Quantitative OCT angiography of the retinal microvasculature and the choriocapillaris in myopic eyes. Invest Ophthalmol Vis Sci.

[31] Fan H, Chen HY, Ma HJ, Chang Z, Yin HQ, Ng DS, et al. Reduced macular vascular density in myopic eyes. Chin Med J (Engl). 2017;130(4):445–51. 10.4103/0366-6999.199844 PMC5324382 28218219

[32] Yang Y, Wang J, Jiang H, Yang X, Feng L, Hu L (2016). Retinal microvasculature alteration in high myopia. Invest Ophthalmol Vis Sci.

[33] Wei WB, Xu L, Jonas JB, Shao L, Du KF, Wang S, et al. Subfoveal choroidal thickness: the Beijing Eye Study. Ophthalmology. 2013;120(1):175–80. 10.1016/j.ophtha.2012.07.048 23009895

[34] Zhang JM, Wu JF, Chen JH, Wang L, Lu TL, Sun W, et al. Macular choroidal thickness in children: the Shandong Children Eye Study. Invest Ophthalmol Vis Sci. 2015;56(13):7646–52. 10.1167/iovs.15-17137 26624496

[35] Jiang R, Wang YX, Wei WB, Xu L, Jonas JB. Peripapillary choroidal thickness in adult chinese: the Beijing Eye Study. Invest Ophthalmol Vis Sci. 2015;56(6):4045–52. 10.1167/iovs.15-16521 26114482

[36] Harb E, Hyman L, Gwiazda J, Marsh-Tootle W, Zhang QH, Hou W (2015). Choroidal thickness profiles in myopic eyes of young adults in the Correction of Myopia Evaluation Trial cohort. Am J Ophthalmol.

[37] Margolis R, Spaide RF (2009). A pilot study of enhanced depth imaging optical coherence tomography of the choroid in normal eyes. Am J Ophthalmol.

[38] Ikuno Y, Kawaguchi K, Nouchi T, Yasuno Y (2010). Choroidal thickness in healthy Japanese subjects. Invest Ophthalmol Vis Sci.

[39] Ding X, Li J, Zeng J, Ma W, Liu R, Li T (2011). Choroidal thickness in healthy Chinese subjects. Invest Ophthalmol Vis Sci.

[40] Mapelli C, Dell'Arti L, Barteselli G, Osnaghi S, Tabacchi E, Clerici M (2013). Choroidal volume variations during childhood. Invest Ophthalmol Vis Sci.

[41] Read SA, Collins MJ, Vincent SJ, Alonso-Caneiro D (2013). Choroidal thickness in childhood. Invest Ophthalmol Vis Sci.

[42] Bidaut-Garnier M, Schwartz C, Puyraveau M, Montard M, Delbosc B, Saleh M (2014). Choroidal thickness measurement in children using optical coherence tomography. Retina.

[43] Nagasawa T, Mitamura Y, Katome T, Shinomiya K, Naito T, Nagasato D (2013). Macular choroidal thickness and volume in healthy pediatric individuals measured by swept-source optical coherence tomography. Invest Ophthalmol Vis Sci.

[44] Park KA, Oh SY (2013). Choroidal thickness in healthy children. Retina.

[45] Ruiz-Moreno JM, Flores-Moreno I, Lugo F, Ruiz-Medrano J, Montero JA, Akiba M (2013). Macular choroidal thickness in normal pediatric population measured by swept-source optical coherence tomography. Invest Ophthalmol Vis Sci.

[46] Read SA, Alonso-Caneiro D, Vincent SJ, Collins MJ (2015). Longitudinal changes in choroidal thickness and eye growth in childhood. Invest Ophthalmol Vis Sci.

[47] Fontaine M, Gaucher D, Sauer A, Speeg-Schatz C (2017). Choroidal thickness and ametropia in children: a longitudinal study. Eur J Ophthalmol.

[48] Xiong S, He X, Zhang B, Deng JJ, Wang JJ, Lv MZ (2020). Changes in choroidal thickness varied by age and refraction in children and adolescents: a 1-year longitudinal study. Am J Ophthalmol.

[49] Troilo D, Nickla DL, Wildsoet CF (2000). Choroidal thickness changes during altered eye growth and refractive state in a primate. Invest Ophthalmol Vis Sci.

[50] Ayhan Z, Kaya M, Ozturk T, Karti O, Oner FH (2017). Evaluation of macular perfusion in healthy smokers by using optical coherence tomography angiography. Ophthalmic Surg Lasers Imaging Retina.

[51] Wilczyński T, Heinke A, Niedzielska-Krycia A, Jorg D, Michalska-Małecka K (2019). Optical coherence tomography angiography features in patients with idiopathic full-thickness macular hole, before and after surgical treatment. Clin Interv Aging.

[52] Milani P, Montesano G, Rossetti L, Bergamini F, Pece A (2018). Vessel density, retinal thickness, and choriocapillaris vascular flow in myopic eyes on OCT angiography. Graefes Arch Clin Exp Ophthalmol.

[53] Mo J, Duan A, Chan S, Wang X, Wei W (2017). Vascular flow density in pathological myopia: an optical coherence tomography angiography study. BMJ Open.

[54] Wu H, Zhang G, Shen M, Xu R, Wang P, Guan Z, et al. Assessment of choroidal vascularity and choriocapillaris blood perfusion in anisomyopic adults by SS-OCT/OCTA. Invest Ophthalmol Vis Sci. 2020. Accepted article. 10.1167/iovs.0.0.31075. 10.1167/iovs.62.1.8 PMC7797932 33393974

[55] Yamashita T, Yamashita T, Shirasawa M, Arimura N, Terasaki H, Sakamoto T (2012). Repeatability and reproducibility of subfoveal choroidal thickness in normal eyes of Japanese using different SD-OCT devices. Invest Ophthalmol Vis Sci.

[56] Matsuo Y, Sakamoto T, Yamashita T, Tomita M, Shirasawa M, Terasaki H (2013). Comparisons of choroidal thickness of normal eyes obtained by two different spectral-domain OCT instruments and one swept-source OCT instrument. Invest Ophthalmol Vis Sci.

[57] Li M, Jin E, Dong C, Zhang C, Zhao M, Qu J (2018). The repeatability of superficial retinal vessel density measurements in eyes with long axial length using optical coherence tomography angiography. BMC Ophthalmol.

[58] Tan CS, Ouyang Y, Ruiz H, Sadda SR (2012). Diurnal variation of choroidal thickness in normal, healthy subjects measured by spectral domain optical coherence tomography. Invest Ophthalmol Vis Sci.

[59] Zhang S, Zhang G, Zhou X, Xu R, Wang S, Guan Z. Changes in choroidal thickness and choroidal blood perfusion in guinea pig myopia. Invest Ophthalmol Vis Sci. 2019;60(8):3074–83. 10.1167/iovs.18-26397 31319419

[60] Zhou X, Zhang S, Zhang G, Chen Y, Lei Y, Xiang J, et al. Increased choroidal blood perfusion can inhibit form deprivation myopia in guinea pigs. Invest Ophthalmol Vis Sci. 2020;61(13):25. 10.1167/iovs.61.13.25 PMC7683853 33211066

